# Dynamic magnetic resonance imaging to quantify pelvic organ prolapse: reliability of assessment and correlation with clinical findings and pelvic floor symptoms

**DOI:** 10.1007/s00192-012-1772-5

**Published:** 2012-04-25

**Authors:** Mariëlle M. E. Lakeman, F. M. Zijta, J. Peringa, A. J. Nederveen, J. Stoker, J. P. W. R. Roovers

**Affiliations:** 1 Department of Obstetrics and Gynaecology, Academic Medical Centre, Room H4-205, PO Box 22700, 1105 DE Amsterdam, The Netherlands; 2 Department of Radiology, Academic Medical Centre, Amsterdam, The Netherlands; 3 Department of Radiology, Onze Lieve Vrouwe Gasthuis, Amsterdam, The Netherlands

**Keywords:** Dynamic MRI, Pelvic organ prolapse, POP-Q, Reference lines

## Abstract

**Introduction and hypothesis:**

The aim of this study was to assess the interobserver agreement of magnetic resonance imaging (MRI)-based staging of pelvic organ prolapse (POP) and to quantify associations between MRI-based POP staging, findings at pelvic examination, and pelvic floor symptoms.

**Methods:**

This was a cross-sectional study of ten symptomatic POP patients, ten symptomatic patients without POP, and ten nulliparous asymptomatic women. Three different observers performed MRI-based POP staging using the pubococcygeal line (PCL), midpubic line (MPL), perineal line, and H line as references.

**Results:**

The interobserver agreement of MRI-based staging of the anterior and middle compartment was good to excellent. In symptomatic women without prolapse, MRI-based and pelvic-examination-based POP staging were poorly correlated. In none of the women were MRI-based POP Quantification (POP-Q) staging and pelvic floor symptoms strongly associated.

**Conclusion:**

The interobserver agreement of MRI-based POP staging is excellent, but the added clinical value of such staging is questionable due to poor association with clinical findings and pelvic floor symptoms.

## Introduction

Pelvic organ prolapse (POP) is a common condition affecting about 30 % of women >40 years old [1–3] and can be associated with micturition and defecation symptoms and sexual dysfunction. Adequate POP staging is important in clinical practice for evaluating symptoms and directing treatment. The most commonly used method for prolapse staging is the Pelvic Organ Prolapse Quantification (POP-Q), as recommended by the International Continence Society (ICS), which is scored during physical examination [4]. Imaging of the pelvic floor might provide important additional anatomical information, as it not only assesses what can be seen on the outside but also provides the internal relationship of pelvic organs. Several imaging techniques have been proposed in addition to the physical examination findings. Defecography is one of the most commonly used techniques. The disadvantage of this technique is that it only evaluates the posterior compartment and is uncomfortable for the patient. In recent years transperineal ultrasound (US) and magnetic resonance imaging (MRI) have been proposed as adjuvant imaging for evaluating all three compartments. Transperineal US has the advantage of easy access and lower costs [5]. However, operator training is required to obtain 3D images and to interpret those images. MRI provides rapid, comprehensive evaluation of the entire pelvis, including support structures and organs [6].

In the literature several reference lines have been proposed to assess POP during dynamic MRI. However, appropriate validation of these reference lines is lacking [7]. Furthermore, the interobserver variability of the method and information as to whether the level of experience of observers affects the assessment are largely unknown. As the most commonly used POP staging method is POP-Q (ICS), POP MRI should be correlated with POP–Q staging [8]. A problem in evaluating POP is that the correlation between anatomical findings on physical examination and pelvic floor symptoms has been reported to be weak [9, 10]. By using dynamic MRI, all three compartments and their mutual relationships can be assessed. Consequently, one might expect improved correlation of pelvic floor symptoms to anatomical abnormalities.

The aim of our study was threefold: The first was to evaluate the reliability of the most commonly proposed reference lines in prolapse staging, as assessed in women with and without POP by three different observers with different levels of experience. The second was to correlate POP as assessed on dynamic MRI with POP as assessed using POP-Q. The third was to evaluate whether MRI staging or POP-Q staging correlates well with pelvic floor symptoms.

## Methods

This prospective study was performed in the AMC, Amsterdam, from 5 January 2010 to 8 December 2010. After approval from the medical ethics committee, three groups of ten women were selected. The first group consisted of women with pelvic floor symptoms and at least stage 2 POP in at least one of the three compartments, as assessed with POP-Q staging. The second group consisted of age-matched controls with bothersome pelvic floor symptoms but with a maximum of stage 1 POP. The third group consisted of nulliparous women without any known anatomical or functional pelvic floor abnormalities. All women gave written informed consent, underwent physical examination and dynamic MRI, and filled out a questionnaire.

### Physical examination

All women underwent a pelvic examination during which prolapse was staged using the POP-Q [4]. Staging was assessed at maximal straining with the patient in the 45° supine position. For correlation between POP-Q and MRI findings, we used the most descending point in every compartment [i.e., anterior (point Ba), middle compartment (point C), and posterior (point Bp)].

### Questionnaires

To assess the presence and discomfort of prolapse symptoms, micturition, or defecation symptoms, women were asked to complete a validated questionnaire. This questionnaire consisted of the Urogenital Distress Inventory (UDI) [11, 12], the Defecation Distress Inventory (DDI) [13], and the Incontinence Impact Questionnaire (IIQ) [11, 12]. The questionnaire also contained several questions such as body mass index (BMI), parity, mode of delivery, and previous gynecological surgery.

### Image acquisition

Each woman underwent MRI in the supine position with her legs parallel and lightly flexed using a 3.0 T MR scanner (Intera, Philips Healthcare, Best, The Netherlands), with a 16-channel phased-array surface coil (SENSE-XL-Torso, Philips Healthcare) centered low on the pelvis for signal reception. No intravenously administered contrast was used, and contrast was administrated to the bladder, urethra, vagina, or rectum. Participants were asked to empty their bladder 1 h prior to the examination. Static MRI for anatomical reference was based on the acquisition of a multishot turbo spin echo (TSE) T2-weighted sequence in axial, coronal, and sagittal planes [field-of-view (FOV) 300 × 300 mm^2^, slice thickness 4 mm, slices 31, slice gap 0.4 mm, TR/TE 3,021/80 ms, in plane resolution of 1.0 × 1.0 mm^2^ ]. Dynamic MRI of the pelvis was performed using a single-shot turbo spin-echo (SSH-TSE) sequence in the midsagittal plane with a temporal resolution of 2 s (FOV 300 × 300 mm^2^, slice thickness 3 mm, TR/TE 2,000/75 ms, 20 dynamics, in-plane resolution of 1.6 × 1.6 mm^2^). Images were obtained at rest and during both maximal contraction of the pelvic floor muscles and maximal straining (Valsalva maneuver). Instructions were given by the technologist prior to each separate series. Patients were instructed to perform the Valsalva maneuver for at least 10 s [14]. This procedure was repeated to acquire the most optimal patient compliance regarding individual contraction and straining outcomes. Average scanning time was 25 min; one MRI cost 300 euros.

### Data analysis

**Fig 1:**
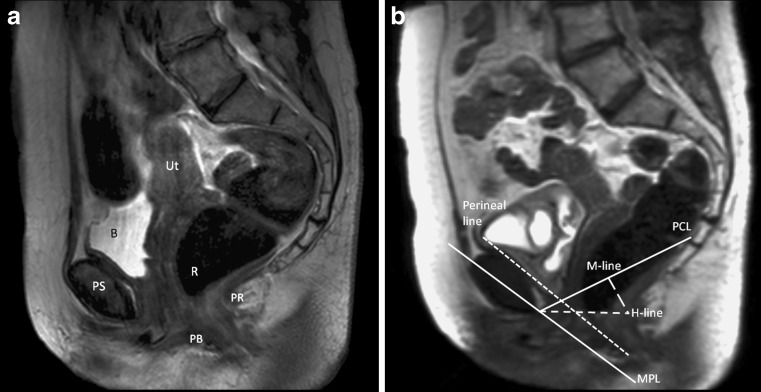
**a** Static T2-weighted turbo spin echo (TSE) at rest for anatomical reference in midsagital plane through the pelvis of a 66-year old woman with pelvic organ prolapse (POP) symptoms. The uterus (Ut), bladder (B), pubic symphysis (PS), rectum (R), puborectal muscle (PR), and perineal body (PB) are shown. **b** Dynamic midsagital half-Fourier-acquisition single-shot turbo spin-echo (HASTE) sequence during straining in the same patient. Reference lines: pubococcygeal line (PCL), H-line, perineal line, midpubic line (MPL), and M-line

The obtained images were evaluated offline by three observers: a radiologist (JP) with 8 years of experience in abdominal radiology, a fourth-year radiology resident (FZ), and a researcher (ML) with no experience in radiology. Images were analyzed both qualitatively and quantitatively using the following reference lines which are also shown in Fig. 1:

- pubococcygeal line (PCL), defined as a straight line between the inferior rim of the pubic bone and the anterior side of the last visible coccygeal joint [6, 15–17];
- H-line, a straight line between the inferior rim of the pubic and posterior wall of the anal canal on the level of the impression of the puborectal sling [6, 15];
- perineal line, a line from the internal surface of the symphysis pubis down to the caudal end of the external anal sphincter [18];
- midpubic line (MPL), a line drawn through the longitudinal axis of the pubic bone and passing through its midequatorial point [18, 19].

Three different pelvic compartments can be identified based on characteristic anatomical landmarks:

- the most posterocaudal point of the bladder base (anterior compartment);
- the most anterocaudal point of the cervix or vaginal vault (middle compartment);
- the most anterocaudal point of the anterior rectal wall (posterior compartment).

The perpendicular distance from the different reference lines to these three different points were assessed. If the anatomical landmark is located above a reference line, the distance has a negative value; below the reference line it has a positive value.

### Statistical analysis

Interobserver reliability was assessed by calculating the intraclass correlation coefficient (ICC) for all four reference lines in the three compartments. An ICC >0.8 indicates excellent agreement, between 0.8 and 0.6 good agreement, between 0.6 and 0.4 moderate agreement, and <0.4 poor agreement [20]. We used Spearman’s rank correlation to correlate POP-Q measurements of the control and POP groups with POP as assessed during dynamic MRI using the four different reference lines. UDI, DDI, and IIQ domain scores, ranging from 0 (no symptoms present) to 100 (all symptoms present and causing maximal bother), were calculated. Spearman’s rank correlation was used to correlate UDI and DDI domain scores with POP-Q measurements and with MRI-staged POP using the PCL line. The PCL was chosen for this comparison because it is the most commonly used reference line and is drawn between two good identifiable points. An exclusion criterion for nulliparous women was the presence of pelvic floor symptoms; therefore, such women were not included in this analysis. We decided not to adjust for multiple testing, as we did not want to miss any important findings [21].

## Results

Table 1 shows patient characteristics, findings on physical examination, and questionnaire results of the three different groups. The prolapse and control groups were comparable in age, BMI, and parity. The prolapse group comprised mainly women with uterine descent or cystocele.

**Table 1:** Patient characteristics

	Nulliparous women	Control group	Prolapse group
Age (mean; SD)	27.7 (3.9)	52.7 (7.4)	58.0 (9.7)
BMI (median; range)	21.5 (20.5–26.3)	22.2 (19.9–31.4)	24.3 (22.2–35.1)
Parity (median; range)	0 (0–0)	2 (0–3)	2 (1–3)
POP-Q stage (median; range)			
Cystocele	0 (0–0)	0 (0–1)	2 (0–3)
Uterine descent	0 (0–0)	0 (0–1)	1 (0–2)
Rectocele	0 (0–0)	1 (0–1)	1 (0–2)
UDI domain scores (mean; SD)			
Overactive bladder	7 (10)	49 (26)	24 (20)
Urinary incontinence	2 (6)	25 (24)	39 (30)
Pain	24 (17)	33 (22)	22 (34)
Genital prolapse	11 (19)	26 (30)	46 (35)
Obstructive micturition	4 (11)	40 (31)	25 (20)
DDI domain scores (mean; SD)			
Constipation	7 (12)	24 (21)	13 (16)
Obstructive defecation	4 (6)	21.9 (21)	19 (16)
Pain	7 (17)	15 (19)	4 (7)
Incontinence	0 (0)	10 (15)	22 (34)
Flatulence incontinence	15 (18)	33 (36)	41 (36)

*POP-Q* Pelvic Organ Prolapse Quantification,* UDI* Urogenital Distress Inventory,* DDI* Defecation Distress Inventory, SD standard deviation, BMI body mass index,

Table 2 shows the interobserver correlation of the four different reference lines as assessed by the three different observers during maximal straining by the study participant. In all three groups of women, a good to excellent correlation was found for anterior and middle compartment assessment using all four reference lines. For the posterior compartment only in the prolapse group, excellent correlations were found using the MPL, H-line, and perineal line. In the nulliparous and control groups, a moderate to good correlation was found in the posterior compartment for the four reference lines. No women had enterocele or intussusception.

**Table 2:** Interobserver correlation [intraclass correlation coefficient (ICC)]: three observers

	Nulliparous		Control group		Prolapse group	
	ICC	95 % CI	ICC	95 % CI	ICC	95 % CI
PCL						
Anterior	0.98	(0.93–0.99)	0.95	(0.83–0.99)	0.95	(0.84–0.99)
Middle	0.94	(0.82–0.98)	0.88	(0.64–0.97)	0.96	(0.89–0.99)
Posterior	0.74	(0.23–0.93)	0.48	(−0.64–0.87)	0.79	(0.34–0.95)
MPL						
Anterior	0.97	(0.92–0.99)	0.82	(0.44–0.96)	0.98	(0.95–0.99)
Middle	0.97	(0.92–0.99)	0.95	(0.84–0.99)	0.95	(0.86–0.99)
Posterior	0.67	(0.02–0.91)	0.91	(0.72–0.98)	0.88	(0.63–0.97)
Perineal line						
Anterior	0.73	(0.20–0.93)	0.57	(−0.47–0.91)	0.98	(0.94–1.0)
Middle	0.96	(0.89–0.99)	0.86	(0.55–0.97)	0.94	(0.82–0.98)
Posterior	0.61	(−0.21–0.91)	0.43	(−0.80–0.86)	0.87	(0.58–0.97)
H-line						
Anterior	0.98	(0.93–0.99)	0.87	(0.60–0.97)	0.98	(0.94–0.99)
Middle	0.85	(0.55–0.96)	0.82	(0.45–0.96)	0.95	(0.86–0.99)
Posterior	0.52	(−0.42–0.87)	0.63	(−0.16–0.91)	0.87	(0.60–0.97)

*CI* confidence interval,* PCL*  pubococcygeal line,* MPL* midpubic line

Table 3 shows the correlation between POP-Q and MRI findings: The prolapse group showed good correlation in the anterior and middle compartments using the perineal line and in the posterior compartment using the PCL and H-lines. The control group showed good correlation in the posterior compartment using the MPL line; the anterior and middle compartments showed poor correlation for the four reference lines. None of the reference lines demonstrated a good correlation in both groups in all three compartments.

**Table 3:** Correlation of the most descending point in the three compartments using Pelvic Organ Prolapse Quantification (POP-Q) staging with the measurements in the same compartment using dynamic magnetic resonance imaging (MRI)

	Control group	Prolapse group
Anterior (Ba)		
PCL	0.22	0.34
MPL	0.11	0.58
Perineal line	0.33	0.64*
H line	0.33	0.39
Middle (C)		
PCL	0.17	0.36
MPL	−0.48	0.54
Perineal line	−0.43	0.67*
H line	−0.61	0.39
Posterior (Bp)		
PCL	0.51	0.79*
MPL	0.70*	0.52
Perineal line	0.62	0.56
H line	0.51	0.84*

Values are correlation coefficients

*PCL* pubococcygeal line,* MPL* midpubic line

*P < 0.05

Table 4 shows the correlation between pelvic floor symptoms as assessed using the UDI and DDI domain scores and prolapse as assessed using the PCL on dynamic MRI and as assessed during physical examination using POP-Q. No significant correlations were found in either group between pelvic floor symptoms and POP-Q findings. Using dynamic MRI in the control group, a significant correlation between the UDI urinary incontinence domain score and prolapse of the middle compartment was found. In the POP group a significant correlation between DDI pain domain score and prolapse of the posterior compartment as assessed using dynamic MRI was found.

**Table 4:** Correlation coefficients between Urogenital Distress Inventory (UDI) and Defecation Distress Inventory (DDI) domain scores and pelvic organ prolapse (POP) as staged using the reference pubococcygeal line (PCL) on dynamic magnetic resonance imaging (MRI) and POP Quantification (POP-Q) staging during physical examination in the control group

	Control group						Prolapse group					
	MRI PCL staging			POP staging			MRI PCL staging			POP staging		
	Anterior	Middle	Posterior	Anterior	Middle	Posterior	Anterior	Middle	Posterior	Anterior	Middle	Posterior
UDI												
Overactive bladder	0.56	0.41	−0.07	0.29	0.22	0.61	0.25	0.06	0.04	−0.07	−0.32	−0.05
Incontinence	0.66	0.77*	0.18	0.06	0.29	0.23	−0.19	0.22	0.18	−0.35	−0.55	0.44
Pain	0.06	−0.22	−0.51	0.39	0.16	0.20	−0.51	0.04	0.03	−0.43	−0.06	0.49
Genital prolapse	0.73	0.28	0.34	−0.08	0.14	0.00	−0.01	0.15	−0.15	−0.03	0.24	0.13
Obstructive micturition	0.03	0.23	−0.36	0.06	−0.05	0.13	0.36	0.28	0.13	0.15	0.15	−0.07
DDI												
Constipation	0.35	0.44	0.51	−0.15	−0.19	0.69	−0.06	0.39	0.48	−0.39	0.23	0.49
Obstructive defecation	0.39	0.39	0.53	−0.22	−0.23	0.50	0.15	0.49	0.78	−0.53	0.27	0.72
Pain	−0.05	−0.14	−0.10	−0.29	−0.35	−0.23	0.52	0.10	0.76*	−0.05	0.16	0.27
Incontinence	0.04	−0.08	−0.04	0.38	0.48	−0.14	−0.04	−0.22	−0.14	0.00	−0.35	0.00
Flatus incontinence	0.43	0.30	0.05	0.46	0.59	0.24	0.46	−0.61	0.05	0.46	0.59	0.24

*UDI* Urogenital Distress Inventory,*DDI* Defecation Distress Inventory,

## Discussion

We found a good to excellent interobserver agreement when evaluating POP on dynamic MRI for the four different reference lines despite the difference in observer experience. In women with pelvic floor symptoms without stage 2 or greater POP, the correlation of dynamic MRI with findings during physical examination was poor overall, whereas in women with at least stage 2 POP, this correlation was moderate. The correlation of dynamic MRI and POP-Q with pelvic floor symptoms was poor for most symptoms in both groups.

Before further interpreting these data, some strengths and weaknesses of our study need to be addressed. First is the relatively low number of women in each group, which may be the reason that we did not observe statistically significant correlations between pelvic floor symptoms and POP-Q findings. The power to show statistically significant correlations might be too limited; however, as the correlation coefficients were very small, we do not think that with more women these correlations would have become statistically significant and clinically relevant. Furthermore, there were several limitations to the method used for obtaining images: First, lying supine and straining does not result in maximum relaxation of the pelvic floor [23]. Although the supine position is not ideal, a study comparing dynamic MRI in the supine position to fluoroscopic cystocolpoproctography in the seated position showed minimal, probably clinically insignificant, differences in POP between these two techniques [22]. Second, unlike during physical examination, during MRI, the patient is not being directly instructed, making coaching on the maximal Valsalva maneuver impossible. Ultimately, this may lead to suboptimal straining results. To pre-empt such problems, patients were coached prior on the outpatient hospital setting on how to perform maximal Valsalva and asked to perform Valsalva for at least 10 s. However, bias due to suboptimal Valsalva maneuvers cannot be excluded. Third, due to the straight position with the legs stretched and close together, it might be more difficult to perform maximum Valsalva maneuver. By repeating the maneuver sequences, an attempt was made to reduce this effect and to achieve a maximum maneuver. Fourth, the plane in which the reference lines are measured might be more posterior than the in vivo plane of the hymen [24]. Furthermore, the plane of the hymen is variable from patient to patient and moves with straining, which might explain the high interobserver variability and poor clinical correlation [23]. Lastly, we chose not to opacify the rectum, which might have caused us to miss minor rectoceles. We avoided this because of the invasive nature and the introduction of gel-bubble artefacts [22, 24].

The strength of this study is that we included women without prolapse on physical examination but with pelvic floor symptoms. This is an interesting group because of the unexplained symptoms; therefore, in this group in particular, MRI might show anatomical abnormalities that were not seen during physical examination. Furthermore, in contrast to previous studies, none of the women underwent previous surgery for prolapse complaints. This makes the relationship between anatomical abnormalities and pelvic floor symptoms more easy to interpret because previous surgery does not alter the anatomical relationship between the three pelvic compartments.

Our first aim was to assess whether POP can be reliably assessed using different reference lines and observers with different levels of experience. In line with previous studies reporting that none of the suggested reference lines is clearly superior, we found an excellent interclass correlation in the anterior and middle compartments no matter what reference line was used [7, 15, 18]. As one of the observers only had a minimal level of experience in viewing MRI, the high intraclass correlation showed that the four reference lines are easy to use, even for observers with a minimal level of experience. In the posterior compartment we found good intraclass correlation in the prolapse group only. An explanation for this might be that in this group of women with obvious anatomical abnormalities, the most descending point is easy to assess, whereas in the group without anatomical abnormalities, such assessment is more difficult. Therefore, assessment might vary more between investigators of the nulliparous and control groups. To date, several studies have reporting on different reference lines and inter- and intraobserver reliability; however, only a few studies reported on correlations between MRI-staged POP and POP-Q-staged POP [18, 19, 24, 25]. Most of those studies describe a poor correlation between clinical and MRI findings and state that POP cannot be described using only one reference line [16, 18, 19]. Our findings are somewhat more optimistic. In the prolapse group we found a good correlation for at least one of the four reference lines in each of the three compartments. However, among the age-matched control group, good correlation was found only in the posterior compartment using the MPL line. This indicates that POP cannot be described by the use of one reference line only and also that the absence of significant prolapse ultimately results in inferior agreement between clinical and MRI findings.

Our second aim was to correlate POP as assessed on MRI with POP-Q findings. We found a poor correlation, probably mainly due to the several mentioned limitations of the imaging method used.

In order to evaluate the additional value of dynamic MRI in explaining pelvic floor symptoms, we evaluated the correlation between prolapse as staged using dynamic MRI and functional complaints scored using validated questionnaires. Only the UDI domain score seemed to correlate well with prolapse of the middle compartment as staged using dynamic MRI. It has been reported that some weakening of the pelvic support mechanisms might increase urinary incontinence symptoms. However, most studies report that with increasing POP, urinary incontinence symptoms decreased due to more obstruction [9, 10]. In our study we also failed to observe a strong correlation between POP and urinary incontinence among the prolapse group, supporting the theory that with increasing POP the correlation between POP and urinary incontinence disappears. Therefore, the clinical relevance of the correlation between the UDI incontinence domain score and POP of the middle compartment using MRI might be limited.

An interesting group in our study is the group of women with pelvic floor symptoms that were not consistent with findings from physical examination. Few previous studies report that pelvic defects were staged more reliable on MRI [25, 26], and it has been suggested that this increased sensitivity of dynamic MRI in staging POP may make it useful for evaluating women with symptoms of pelvic floor relaxation who have negative findings on clinical examination using POP-Q [26]. However, in our study, the correlation of symptoms with POP as staged using dynamic MRI and POP-Q was moderate at best for all DDI domain scores and for most UDI domain scores. Consequently, based on our data, the additional diagnostic value of dynamic MRI for staging POP, even in women with unexplained symptoms, seems limited. However, the additional value of dynamic MRI might be that its multiplanar representation has the potential to yield much more anatomic information about the pelvic floor musculature and other support structures that might explain more of the functional complaints.

The results we found on the added value of MRI in evaluating POP must be compared with other imaging techniques such as transperineal US. This technique has a higher temporal and spatial resolution and a good correlation with POP-Q staging [27] and with the symptom of feeling a vaginal lump [28]. Furthermore considering the easy access and lower costs, this technique may be more valuable in clinical practice. However, dynamic MRI has the advantage of a larger FOV and therefore allows visualization of a larger volume of the pelvic floor compared with transperineal US. Therefore, MRI might have an added value mainly in research areas considering its capability to evaluate the entire pelvis, including support structures and organs [6, 29].

## Conclusion

In this study all four evaluated reference lines for assessing POP using dynamic MRI have an excellent interobserver correlation, even if used by less experienced viewers. In line with previous findings, no reference line could be identified that optimally correlated with finding on clinical examination in all three compartments among women with and without stage 2 or greater POP. Also, the correlation with clinical symptoms is not satisfying. In general, we question the added value of dynamic MRI in assessing anatomical abnormalities involved in POP.
